# Fecal metabolomic profiles by LDL-C target achievement status in statin-treated patients: a cross-sectional study

**DOI:** 10.1016/j.jlr.2026.101136

**Published:** 2026-09-01

**Authors:** Yi-Hsueh Liu, Wei-Chun Hung, Ping-Hsun Wu, Po Peng, Pei-Yu Wu, Jiun-Chi Huang, Yi-Hsiung Lin, Szu-Chia Chen

**Affiliations:** 1 Graduate Institute of Clinical Medicine, College of Medicine, Kaohsiung Medical University, Kaohsiung, Taiwan; 2 Department of Internal Medicine, Kaohsiung Municipal Siaogang Hospital, Kaohsiung Medical University Hospital, Kaohsiung Medical University, Kaohsiung, Taiwan; 3 Division of Cardiology, Department of Internal Medicine, Kaohsiung Medical University Hospital, Kaohsiung Medical University, Kaohsiung, Taiwan; 4 Faculty of Medicine, College of Medicine, Kaohsiung Medical University, Kaohsiung, Taiwan; 5 Department of Microbiology and Immunology, College of Medicine, Kaohsiung Medical University, Kaohsiung, Taiwan; 6 Division of Nephrology, Department of Internal Medicine, Kaohsiung Medical University Hospital, Kaohsiung Medical University, Kaohsiung, Taiwan; 7 Welgene Biotech. Co., Ltd., Taipei, Taiwan; 8 Center for Lipid Biosciences, Department of Medical Research, Kaohsiung Medical University Hospital, Kaohsiung Medical University, Kaohsiung, Taiwan; 9 Research Center for Precision Environmental Medicine, Kaohsiung Medical University, Kaohsiung, Taiwan

**Keywords:** statin, low-density lipoprotein cholesterol, microbiota, fecal metabolomics

## Abstract

Whether the fecal metabolome differs according to intensive low-density lipoprotein cholesterol (LDL-C) target achievement among statin-treated patients is unclear. In this cross-sectional study, 124 statin-treated adults with chronic disease were stratified by fasting LDL-C into a target-achieved group (<70 mg/dl, n = 52) and a target-not-achieved group (≥70 mg/dl, n = 72). Stool samples were profiled by untargeted ultra-high-performance liquid chromatography–tandem mass spectrometry, and multivariable models adjusted for age, sex, chronic kidney disease, and angiotensin-converting enzyme inhibitor/angiotensin receptor blocker use were used to identify metabolites independently associated with target achievement. Statin dose, treatment duration, and glucose-lowering therapy were also compared between the groups. Paired 16S rRNA gene sequencing data available for a subset (n = 86) were used for integrative correlation and network analyses. Partial least-squares discriminant analysis showed separation between the two groups. Eight annotated metabolites—glutamine, glutamate, phenylalanine, N-acetyl-L-phenylalanine, L-methionine, N-acetyl-L-methionine, lysine, and N-methyl-D-aspartic acid, predominantly amino acids and their derivatives—were present at lower fecal levels in participants who achieved the LDL-C target. Metabolite set enrichment analysis implicated amino acid and nitrogen metabolism, and multiomics network analysis identified an *Anaerotruncus*-centered amino acid module with high degree centrality. In conclusion, LDL-C target achievement under statin therapy was associated with a coherent “low fecal amino acid” signature and an *Anaerotruncus*-linked microbe–metabolite hub.These findings suggest that intestinal nutrient handling and gut microbial amino acid metabolism may contribute to variability in LDL-C response, and they warrant prospective mechanistic evaluation.

Statin therapy is well-established as a cornerstone in the prevention of atherosclerotic cardiovascular disease, primarily by lowering low-density lipoprotein cholesterol (LDL-C) levels (1). A large-scale meta-analysis demonstrated that each ∼1 mmol/L (∼39 mg/dl) reduction in LDL-C was associated with approximately a 20% reduction in major vascular events, and that more intensive LDL-C lowering was associated with further risk reductions with no clear lower threshold (1). Accordingly, clinical guidelines stratify LDL-C goals according to absolute atherosclerotic risk. Contemporary recommendations reserve the most intensive goal of LDL-C <55 mg/dl for very high-risk patients with established atherosclerotic cardiovascular disease, whereas LDL-C <70 mg/dl remains the recommended threshold for high-risk individuals in whom risk is conferred principally by diabetes, chronic kidney disease (CKD) or a comparable burden of risk factors rather than by prior atherosclerotic events (2, 3, 4). Beyond their lipid-lowering efficacy, statins exert pleiotropic effects such as enhancing endothelial function, attenuating inflammatory responses, affecting antiplatelets, and stabilizing atherosclerotic plaques (5). These benefits underscore why statins remain the first-line therapy for dyslipidemia and cardioprotection (2, 3, 4).

The human gut microbiome is now recognized as an important modulator of host metabolism and drug response. Medications can alter the composition and function of the intestinal microbiota, and conversely, gut microbes can metabolize drugs or modify host pathways that affect drug efficacy (6). Increasing evidence suggests a bidirectional relationship between statins and the gut ecosystem, and that statin therapy can remodel the gut microbiota (6). For example, a large cohort study found that statin-treated individuals had a distinct fecal microbiota profile with a significantly lower prevalence of a dysbiotic enterotype associated with obesity-related inflammation (7). These drug-induced microbial changes extend to microbial metabolism, and recent studies have shown that statin therapy can perturb the gut microbiota and fecal metabolome, notably altering bile acid profiles and short-chain fatty acid (SCFA) production (6, 8, 9). In both patient and animal models, statins have been shown to increase fecal levels of primary bile acids (e.g. chenodeoxycholic acid) while depleting certain secondary bile acids such as ursodeoxycholate, likely by suppressing the gut bacteria (such as *Clostridium* spp.) involved in bile acid biotransformation (6, 10). Such changes have functional implications; for example, statin-induced changes in microbiota and bile acids have been linked to lower intestinal glucagon-like peptide-1 levels and worsened glycemic control in experimental settings (10). This growing body of evidence underscores a bidirectional relationship between statins and the gut microbiome, wherein statins can reshape the gut microbial community and its metabolites, which in turn may influence host metabolism.

Metabolomics has emerged as a powerful approach to investigate host-microbiome interactions and identify biochemical markers of drug response (11, 12). Unlike genomics or proteomics, metabolomic profiling captures a real-time snapshot of the small-molecule metabolites that reflect both host and microbial activity (13). This comprehensive analysis of the “chemical fingerprint” can provide an insight into metabolic pathways beyond that provided by traditional lipid measurements (14, 15). For example, metabolomic investigations have revealed novel risk-related metabolites in cardiovascular disease, and a landmark study identified gut microbial products of dietary choline—namely trimethylamine N-oxide (TMAO) and its precursors—as predictors of incident cardiovascular events, linking gut flora metabolism with atherosclerosis risk (15). These findings illustrate how metabolomic analysis can be used to identify changes in pathways (e.g., in microbial nutrient metabolism) that would be missed by focusing only on canonical risk factors such as LDL-C. Notably, most previous pharmacometabolomic studies on statins have focused on plasma or serum metabolites (16, 17, 18), whereas the fecal metabolome, which reflects gut microbial functional output and unabsorbed dietary compounds, remains comparatively underexplored in the context of lipid-lowering therapy (10, 19). Fecal metabolomics offers a unique window into host–microbe co-metabolism in the gut environment. By analyzing stool samples, it is possible to capture alterations in microbial fermentation products, bile acid transformations, and other gut-derived metabolites that may mediate or indicate an individual’s response to statin treatment (19, 20). Key microbial metabolites such as bile acids, SCFAs, and possibly tryptophan-derived indoles or other amino acid catabolites may act as biochemical modulators of cholesterol metabolism and vascular health (12). Identifying these metabolites could thus provide insights beyond traditional blood lipid measurements, offering potential biomarkers of statin response and new therapeutic targets.

Despite these insights, most previous studies have examined differences in the gut microbiome or metabolites between statin users and non-users, rather than between statin users stratified by LDL-C goal attainment. It remains unclear whether statin users who achieve an intensive LDL-C target (e.g. <70 mg/dl) have a distinct gut metabolic profile compared to those who do not achieve the target. Therefore, in this cross-sectional study, we profiled the fecal metabolome of statin-treated patients with chronic diseases (including hypertension, diabetes, or CKD), and compared those who achieved LDL-C <70 mg/dl with those who did not. Using untargeted liquid chromatography-mass spectrometry (LC-MS) analysis of stool samples, we aimed to identify metabolites associated with stringent LDL-C control. We hypothesized that those with well-controlled LDL-C would have a unique fecal metabolomic signature potentially reflecting alterations in microbial amino acid metabolism and other pathways, and that this may provide insights into the role of the gut microbiota in optimal statin response and cardiovascular risk reduction.

## Materials and methods

### Study participants

Between March and July 2020, we enrolled 124 outpatients with chronic diseases (73 men and 51 women) from a regional hospital in southern Taiwan. In this study, we defined patients with a chronic disease as those with hypertension, diabetes, or CKD who had been receiving ongoing treatment for more than three months. Individuals aged 20–99 years were eligible. All of the patients were receiving statin treatment. The exclusion criteria were: active malignancy, abdominal cancer, a history of abdominal radiotherapy, acute or chronic inflammatory conditions, or the use of prescription antibiotics within the three months preceding enrollment. The study protocol was approved by the Institutional Review Board of Kaohsiung Medical University Hospital (KMUHIRB-G(II)-20190014; approval date: May 28, 2019). Written informed consent was obtained from all participants, and all procedures were conducted in accordance with the principles of the Declaration of Helsinki and applicable guidelines and regulations.

### Demographic, medical, and laboratory data

Data on demographic characteristics (age, sex and body mass index [BMI]), comorbidities, medication exposure, and laboratory measurements were extracted from electronic health records. Hypertension was defined as systolic blood pressure ≥ 140 mmHg, diastolic blood pressure ≥ 90 mmHg, or current use of antihypertensive therapy. Diabetes was defined as glycated hemoglobin (HbA1c) ≥ 6.5% or treatment with antidiabetic agents. CKD was defined by evidence of kidney damage or an estimated glomerular filtration rate (eGFR) <60 ml/min/1.73 m^2^ for at least three months. After an overnight fast, venous blood was collected for biochemical profiling, including fasting glucose, HbA1c, triglycerides, total cholesterol, high-density lipoprotein cholesterol (HDL-C), LDL-C, hemoglobin, albumin, aspartate aminotransferase, and alanine aminotransferase. The four-variable Modification of Diet in Renal Disease (MDRD) equation was used to calculate eGFR. Statin therapy comprised atorvastatin, rosuvastatin, and a niacin-lovastatin combination. Because alternate-day regimens are common in routine practice in Taiwan, statin exposure was expressed as the average daily dose (labelled tablet strength × tablets per administration × a dosing-frequency factor: once daily = 1, twice daily = 2, every other day = 0.5, every 4 days = 0.25, once weekly = 1/7). To allow comparison across agents, this was converted to an atorvastatin-equivalent daily dose (atorvastatin × 1, rosuvastatin × 2, lovastatin × 0.25), based on the published equipotency of atorvastatin 10 mg, rosuvastatin 5 mg and lovastatin 40 mg daily (4, 21, 22, 23). Treatment duration was ascertained from prescription records over a 5-years look-back window from stool collection, with gaps of ≤ 30 days regarded as continuous treatment and >90 days as an interruption; all participants had received statin therapy continuously for at least 3 months at a stable agent and dose before sampling. Data on concomitant medications including angiotensin-converting enzyme inhibitors (ACEIs)/angiotensin-receptor blockers (ARBs), beta-blockers, calcium channel blockers, diuretics, fibrates, aspirin, and ezetimibe were also recorded. Glucose-lowering therapy was classified as insulin and eight oral drug classes (biguanide, sulfonylurea, meglitinide, dipeptidyl peptidase-4 [DPP-4] inhibitor, sodium–glucose cotransporter-2 [SGLT2] inhibitor, thiazolidinedione, α-glucosidase inhibitor, and glucagon-like peptide-1 [GLP-1] receptor agonist), each component of a fixed-dose combination being counted separately. The participants were categorized by fasting LDL-C target attainment into two groups: the target-achieved group (LDL-C <70 mg/dl) and the target-not-achieved group (LDL-C ≥70 mg/dl) (Table 1).

**Table 1:** Clinical characteristics of the study population

Characteristics	LDL-C Target–achieved group LDL-C <70 mg/dl (n = 52)	LDL-C target–not-achieved group LDL-C ≥ 70 mg/dl (n = 72)	*p*	All Patients (n = 124)
Age (year)	67.4 ± 9.1	65.2 ± 11.2	0.236	66.1 ± 10.4
Male (%)	58%	60%	0.821	59%
BMI (kg/m^2^)	26.6 ± 3.5	26.2 ± 4.3	0.663	26.4 ± 4.0
Comorbidities				
Diabetes mellitus (%)	88%	86%	0.700	87%
Hypertension (%)	79%	71%	0.314	74%
Cardiovascular disease (%)	12%	13%	0.871	12%
Chronic kidney disease (%)	46%	53%	0.467	50%
Lipid-lowering therapy				
Atorvastatin (%)	26 (50.0%)	31 (43.1%)	0.444	57 (46.0%)
Rosuvastatin (%)	26 (50.0%)	38 (52.8%)	0.760	64 (51.6%)
Niacin and lovastatin combination (%)	0 (0.0%)	3 (4.2%)	0.264	3 (2.4%)
Ezetimibe (%)	1 (1.9%)	2 (2.8%)	1.000	3 (2.4%)
Statin average daily dose, n (%)				
Atorvastatin <10 mg	3 (5.8%)	0 (0.0%)	0.071	3 (2.4%)
Atorvastatin 10 mg	4 (7.7%)	10 (13.9%)	0.282	14 (11.3%)
Atorvastatin 20 mg	14 (26.9%)	16 (22.2%)	0.546	30 (24.2%)
Atorvastatin 40 mg	5 (9.6%)	5 (6.9%)	0.741	10 (8.1%)
Rosuvastatin <5 mg	3 (5.8%)	6 (8.3%)	0.733	9 (7.3%)
Rosuvastatin 5 mg	8 (15.4%)	23 (31.9%)	0.036	31 (25.0%)
Rosuvastatin 10 mg	15 (28.8%)	9 (12.5%)	0.023	24 (19.4%)
Lovastatin 40 mg (with niacin)	0 (0.0%)	3 (4.2%)	0.264	3 (2.4%)
Dosing frequency, n (%)				
Once daily	35 (67.3%)	34 (47.2%)	0.026	69 (55.6%)
Every other day or less frequent	17 (32.7%)	38 (52.8%)	0.026	55 (44.4%)
Atorvastatin-equivalent daily dose (mg)	17.9 ± 9.2	15.1 ± 8.6	0.086	16.3 ± 8.9
Duration of statin therapy (months)	35.2 ± 25.8	43.1 ± 26.9	0.101	39.8 ± 26.6
Categories, n (%)				
<1 year	13 (25.0%)	10 (13.9%)	0.116	23 (18.5%)
1 to < 3 years	15 (28.8%)	21 (29.2%)	0.969	36 (29.0%)
3 to < 5 years	16 (30.8%)	20 (27.8%)	0.717	36 (29.0%)
≥ 5 years	8 (15.4%)	21 (29.2%)	0.074	29 (23.4%)
Other medication				
ACEI/ARB (%)	81%	60%	0.013	69%
Beta-blocker (%)	25%	22%	0.718	23%
CCB (%)	54%	44%	0.301	48%
Diuretic (%)	27%	26%	0.947	27%
Fibrates (%)	6%	7%	1.000	6%
Aspirin (%)	12%	18%	0.320	15%
Glucose-lowering therapy				
Insulin, n (%)	12 (23.1%)	10 (13.9%)	0.186	22 (17.7%)
Oral hypoglycemic agent (any), n (%)	46 (88.5%)	56 (77.8%)	0.124	102 (82.3%)
Biguanide (metformin), n (%)	28 (53.8%)	33 (45.8%)	0.378	61 (49.2%)
Sulfonylurea, n (%)	25 (48.1%)	37 (51.4%)	0.716	62 (50.0%)
Meglitinide, n (%)	5 (9.6%)	4 (5.6%)	0.490	9 (7.3%)
Dipeptidyl peptidase-4 inhibitor, n (%)	22 (42.3%)	20 (27.8%)	0.092	42 (33.9%)
Sodium–glucose cotransporter-2 inhibitor, n (%)	5 (9.6%)	14 (19.4%)	0.134	19 (15.3%)
Thiazolidinedione, n (%)	5 (9.6%)	4 (5.6%)	0.492	9 (7.3%)
α-Glucosidase inhibitor, n (%)	10 (19.2%)	16 (22.2%)	0.686	26 (21.0%)
Glucagon-like peptide-1 receptor agonist, n (%)	0 (0.0%)	0 (0.0%)	—	0 (0.0%)
Clinical laboratory data				
Fasting glucose (mg/dl)	127.3 ± 29.2	135.8 ± 32.2	0.134	132.2 ± 31.1
HbA_1c_ (%)	7.03 ± 1.08	7.28 ± 1.25	0.239	7.17 ± 1.18
eGFR (mL/min/1.73 m^2^)	50.8 ± 22.6	49.4 ± 24.7	0.734	50.0 ± 23.8
Triglyceride (mg/dl)	114.5 (75.8-171.8)	111.0 (82.3-147.0)	0.704	111.5 (81.0-154.0)
Total cholesterol (mg/dl)	138.0 ± 24.5	178.3 ± 39.2	<0.001	161.4 ± 39.2
HDL-C (mg/dl)	44.5 ± 10.4	48.0 ± 12.2	0.092	46.5 ± 11.6
LDL-C (mg/dl)	55.8 ± 9.9	93.7 ± 22.3	<0.001	77.8 ± 26.1
Hemoglobin (g/dl)	12.5 ± 1.7	13.1 ± 2.2	0.085	12.9 ± 2.0
Albumin (g/dl)	4.18 ± 0.24	4.10 ± 0.25	0.086	4.13 ± 0.25
Aspartate Transaminase (IU/L)	31.6 ± 42.1	27.4 ± 13.2	0.440	29.1 ± 28.7
Alanine Transaminase (IU/L)	31.4 ± 54.7	26.1 ± 18.8	0.447	28.3 ± 38.1

Data are expressed as mean ± standard deviation or median (interquartile range) for continuous variables and as number (percentage) for categorical variables. Between-group comparisons were performed using the independent *t* test or Mann–Whitney U test for continuous variables. Categorical variables were compared using the Pearson chi-square test when all expected cell counts were ≥ 5 and Fisher's exact test otherwise.

Because alternate-day and other non-daily statin regimens are common in routine practice in Taiwan, statin exposure is reported as the average daily dose rather than the dispensed tablet strength. The average daily dose was calculated as labelled tablet strength × number of tablets per administration × dosing-frequency factor (once daily = 1, twice daily = 2, every other day = 0.5, every 4 days = 0.25, once weekly = 1/7). To allow comparison across agents, the average daily dose was further converted to an atorvastatin-equivalent daily dose using published LDL-C–lowering equipotency factors (atorvastatin × 1, rosuvastatin × 2, lovastatin × 0.25), under which atorvastatin 10 mg, rosuvastatin 5 mg and lovastatin 40 mg daily are equipotent; for example, rosuvastatin 10 mg once daily corresponds to 20 mg and atorvastatin 40 mg every other day to 20 mg of atorvastatin equivalents. The *P* value shown is from the independent *t* test (Levene *P* = 0.803); the corresponding median (interquartile range) was 20.0 (10.0–20.0) versus 10.0 (10.0–20.0) mg (Mann–Whitney U test, *P* = 0.045).

Duration of statin therapy was ascertained from outpatient prescription records using a 5-year look-back window from the date of stool collection; prescription gaps of ≤ 30 days were regarded as continuous treatment and gaps of > 90 days as an interruption. Participants still receiving statin therapy at the beginning of the look-back window were classified as ≥ 5 years. All participants had been receiving statin therapy continuously for at least 3 months at a stable agent and dose before stool collection.

ACEI, angiotensin-converting enzyme inhibitor; ARB, angiotensin receptor blocker; BMI, body mass index; CCB, calcium channel blocker; eGFR, estimated glomerular filtration rate; HbA1c, glycated hemoglobin; HDL-C, high-density lipoprotein cholesterol; LDL-C, low-density lipoprotein cholesterol.

### Study cohort and sample collection

A total of 124 participants were enrolled in this study to investigate the fecal metabolomic signatures associated with LDL-C therapeutic response, of whom 52 were in the target-achieved group and 72 in the target-not-achieved group. All 124 participants underwent fecal metabolomic profiling, and paired 16S rRNA microbiome data were obtained from a subset of 86 participants (LDL-C <70 mg/dl, n = 37; LDL-C ≥ 70 mg/dl, n = 49) for integrative multiomics analysis (Fig. 1).

**Fig. 1:**
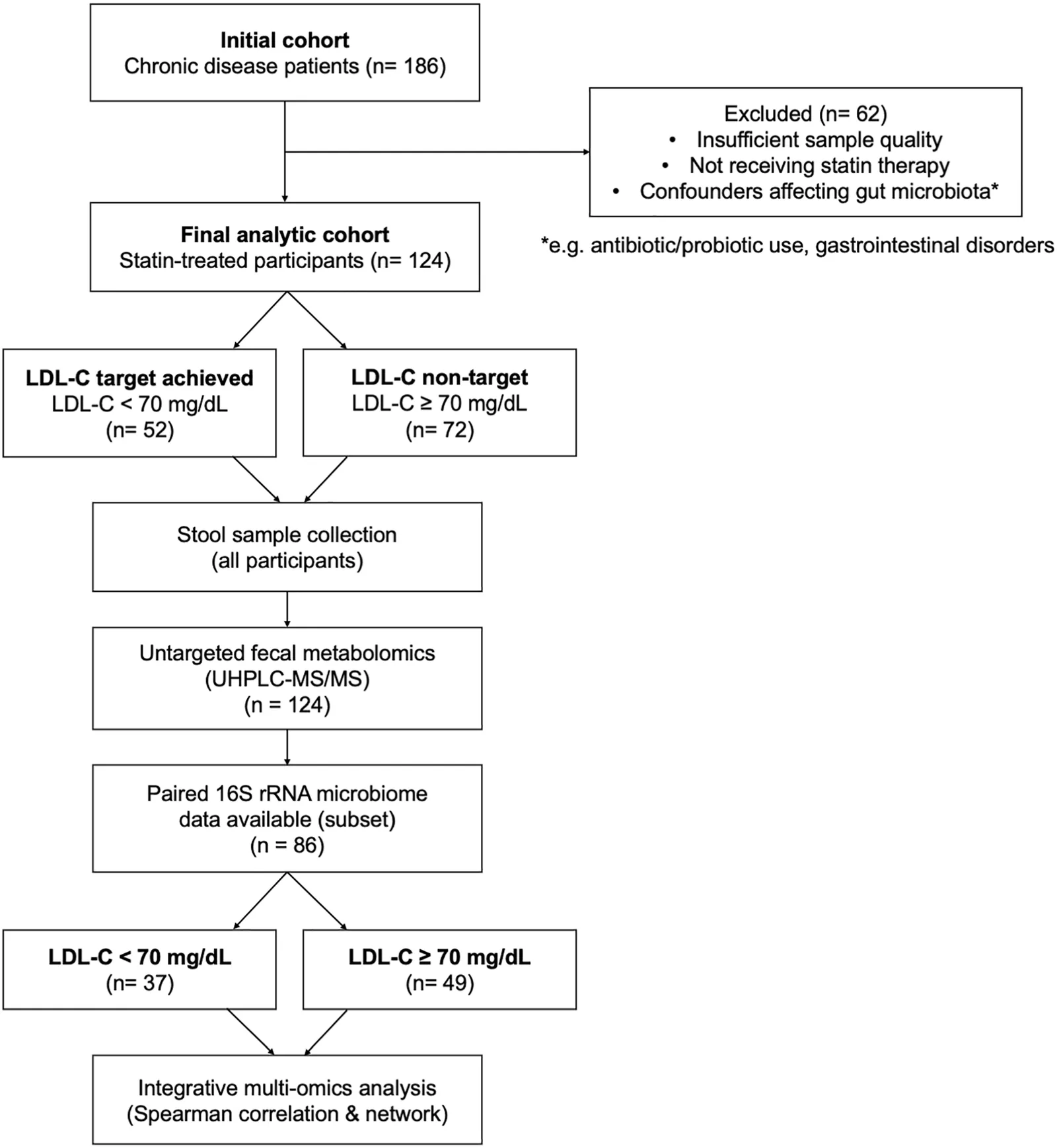
Flowchart of cohort selection and multiomics analytical strategy. From an initial cohort of 186 patients with chronic diseases, 62 individuals were excluded because they were not receiving statin therapy, had confounding factors affecting the gut microbiota (e.g., antibiotic/probiotic use, gastrointestinal disorders), or had insufficient sample quality. The final analytic cohort consisted of 124 statin-treated participants, categorized into the LDL-C target-achieved group (LDL-C <70 mg/dl, n = 52) and target-not-achieved group (LDL-C ≥ 70 mg/dl, n = 72). All 124 included participants provided stool samples for metabolomic phenotyping (UHPLC-MS/MS). A subset of 86 participants (LDL-C <70 mg/dl, n = 37; LDL-C ≥ 70 mg/dl, n = 49) also provided paired 16S rRNA microbiome data for integrative multiomics analysis. The figure illustrates the stepwise exclusion process, group stratification criteria, and downstream integration of clinical and multiomics datasets.

### Stool sample preparation

Fecal samples were processed using a systematic extraction protocol (LWHK). Approximately 30 mg of lyophilized fecal material was homogenized in 750 μl of ice-cold water (Eppendorf Thermomixer, 1000 rpm, 5 min), followed by 20 min of ultrasonication. After centrifugation at 15,000 rcf (4°C, 10 min), the pellets underwent a second extraction with 750 μl of ice-cold methanol (MeOH) containing internal standards. The resulting supernatants were combined and diluted fourfold with MeOH before LC-MS analysis.

### Untargeted metabolomics with UHPLC-MS/MS

Metabolomic profiling was performed on a Dionex Ultimate 3000 UHPLC coupled with a Q Exactive Plus quadrupole–Orbitrap mass spectrometer (Thermo Fisher Scientific). Separation was achieved using an ACQUITY Premier HSS T3 column (2.1 × 100 mm, 1.8 μm) at 40°C. Mobile phases consisted of (A) water and (B) acetonitrile, both with 0.1% formic acid (flow rate: 0.3 ml/min). Detection was operated in ESI ± mode (mass range: 80–1200 m/z; resolution: 70,000 MS1, 17,500 MS2). A data-dependent acquisition strategy was employed. Pooled quality control (QC) samples were injected every 10 study samples to monitor stability.

### Data preprocessing and metabolite annotation

Raw data were processed using Compound Discoverer v3.3.0 for peak extraction and alignment. Features were retained if the coefficient of variation across QC samples was <30%. Annotation involved matching accurate mass (<5 ppm), isotopic patterns, and MS/MS fragments against the LWHK internal database and public resources (mzCloud, ChemSpider). Identified metabolites were categorized by confidence level (Level 1: confirmed; Level 2: MS/MS match).

### Microbiome profiling and multiomics integration

Bacterial abundances (16S rRNA) were aggregated at the genus level and normalized using total sum scaling. Integrative analysis (Spearman correlation and network) was restricted to the paired sample subset (n = 86) to ensure biological correspondence.

### Statistical analysis and network topology

Statistical significance for univariate comparisons was defined as a false discovery rate (FDR)-adjusted *P* value (*q*) <0.05. Multivariate analyses (principal component analysis [PCA] and partial least-squares discriminant analysis [PLS-DA]) were conducted using LWHK OmicsViz. Metabolic features were prioritized based on a variable importance in projection (VIP) score >1.4 and |*log*_*2*_Fold Change| >1. We used MaAsLin2 (24) to identify metabolites independently associated with LDL-C achievement, adjusting for age, sex, CKD status, and ACEI/ARB treatment. Using the default parameters for high-dimensional omics data, an FDR–corrected *q*-value threshold of <0.25 was applied to determine statistical significance. Pathway enrichment was performed using MetaboAnalyst 6.0. Interaction networks were constructed using a threshold of *|r|* >0.35 and visualized with convex hull delineation to define functional hubs. To assess whether statin exposure rather than LDL-C target achievement could account for the observed differences, the correlation between the atorvastatin-equivalent daily dose and LDL-C was examined by Spearman’s rank correlation.

## Results

### Study population and clinical characteristics

A total of 124 statin-treated participants were included, including 52 in the LDL-C target-achieved group (LDL-C <70 mg/dl) and 72 in the target-not-achieved group (LDL-C ≥ 70 mg/dl) (Fig. 1 and Table 1). The two groups were similar in age, sex distribution, and BMI. Comorbidity profiles were also comparable between the two groups, including diabetes mellitus, hypertension, cardiovascular disease, and CKD. The distribution of statin agents was also similar (atorvastatin 50.0% versus 43.1%, *P* = 0.444; rosuvastatin 50.0% versus 52.8%, *P* = 0.760), as was ezetimibe use (1.9% versus 2.8%, *P* = 1.000).

Among the average daily dose categories, only rosuvastatin 5 mg (15.4% versus 31.9%, *P* = 0.036) and 10 mg (28.8% versus 12.5%, *P* = 0.023) differed, whereas all atorvastatin strata were comparable; once-daily administration was more frequent in the target-achieved group (67.3% versus 47.2%, *P* = 0.026), reflecting the common use of alternate-day regimens in local practice. The atorvastatin-equivalent daily dose was only marginally higher in the target-achieved group (17.9 ± 9.2 versus 15.1 ± 8.6 mg, *P* = 0.086) and was not correlated with LDL-C (Spearman ρ = −0.146, *P* = 0.107). The duration of statin therapy did not differ (35.2 ± 25.8 versus 43.1 ± 26.9 months, *P* = 0.101), nor did its categorical distribution (Table 1).

Glucose-lowering therapy did not differ between the groups. The proportions receiving insulin (23.1% versus 13.9%, *P* = 0.186) and any oral hypoglycemic agent (88.5% versus 77.8%, *P* = 0.124) were comparable, as were those receiving each of the eight oral drug classes, among which the lowest *P* value was 0.092 (DPP-4 inhibitors). Metformin was prescribed to 53.8% of the target-achieved group and 45.8% of the target-not-achieved group (*P* = 0.378), and no participant received a GLP-1 receptor agonist (Table 1).

Regarding concomitant medications, the use of ACEIs/ARBs was higher in the LDL-C target-achieved group than in the target-not-achieved group (81% versus 60%, *P* = 0.013), however, there was no significant difference in the other medications. As expected, lipid profiles differed by group, and total cholesterol and LDL-C levels were lower in the LDL-C target-achieved group than in the target-not-achieved group (total cholesterol: 138.0 ± 24.5 versus 178.3 ± 39.2 mg/dl, *P* < 0.001; LDL-C: 55.8 ± 9.9 versus 93.7 ± 22.3 mg/dl, *P* < 0.001). HDL-C and triglyceride levels were similar between the two groups. No significant between-group differences were observed in fasting glucose, HbA1c, eGFR, hemoglobin, albumin, aspartate transaminase, or alanine transaminase (Table 1).

### Global fecal metabolomic patterns and differential fecal metabolites by LDL-C target achievement

Differences in global fecal metabolomic patterns were evaluated using PCA and PLS-DA. Unsupervised PCA demonstrated high analytical stability through tight QC clustering (Fig. 2A). Supervised PLS-DA showed a distinct separation between the LDL-C target-achieved and target-not-achieved groups (Fig. 2B).

**Fig. 2:**
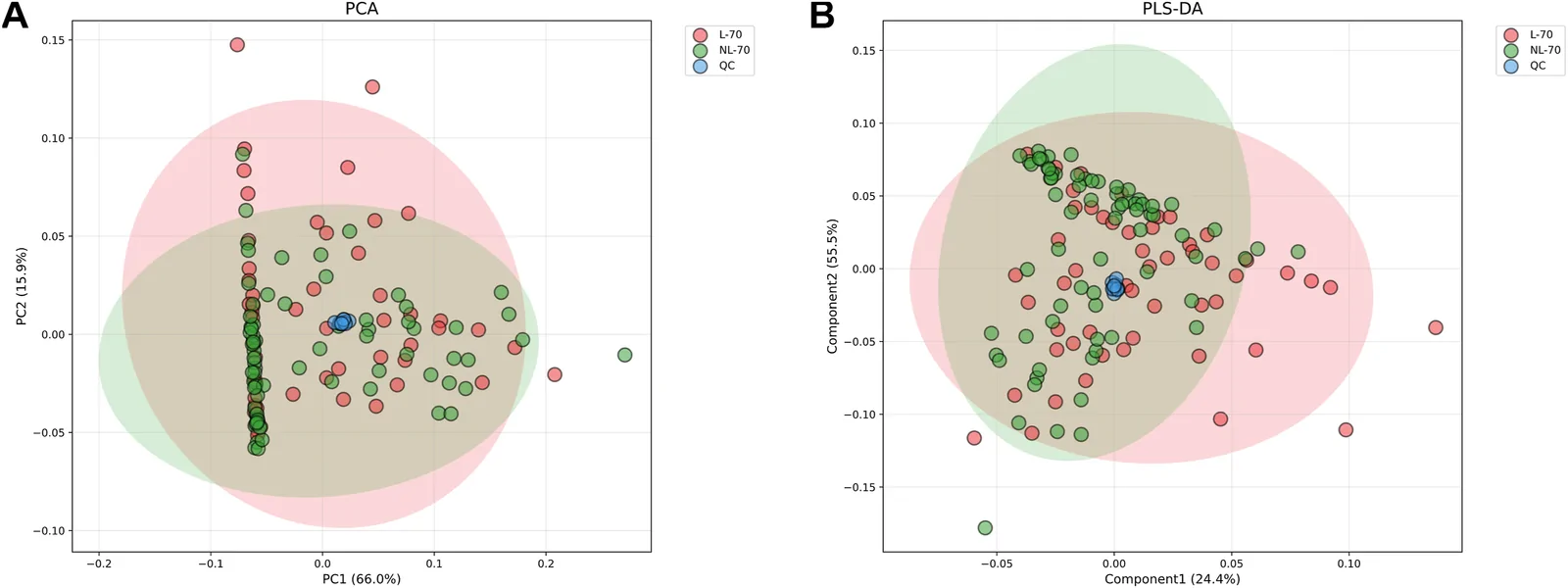
Global metabolomic profiling of the patients. A: Principal component analysis (PCA) score plot showing the distribution of samples based on untargeted metabolomic features (PC1 = 66.0%, PC2 = 15.9%). B: Partial least squares–discriminant analysis (PLS-DA) score plot maximizing the separation between the LDL-C target-achieved (L-70, red) and target-not-achieved (NL-70, green) groups (Component 1 = 24.4%, Component 2 = 55.5%). Blue dots represent pooled QC samples, demonstrating instrument stability. Ellipses represent 95% confidence regions.

Volcano plot analysis identified differential metabolite features between the LDL-C target-achieved and target-not-achieved groups (Fig. 3). Using nominal thresholds of *P* < 0.05 and |log2(fold change)| >1, 118 features were upregulated, and 21 features were downregulated in the LDL-C target-achieved group, while 8047 features were not significant (Fig. 3).

**Fig. 3:**
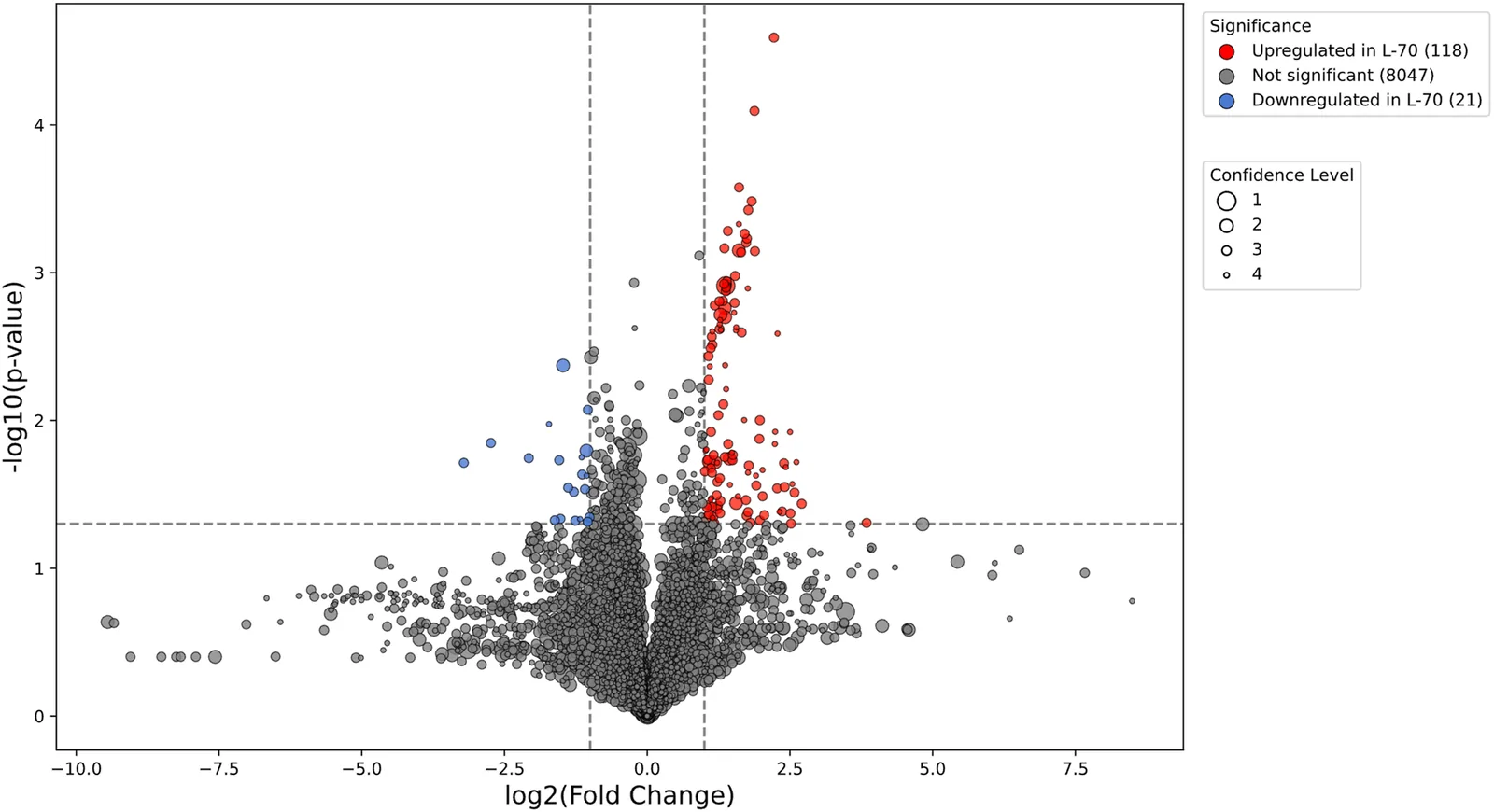
Differential metabolomic profiles between the LDL-C target-achieved (LDL-C <70 mg/dl) and target-not-achieved groups (LDL-C ≥ 70 mg/dl). The volcano plot highlights 139 significantly altered features based on thresholds of *P* < 0.05 and |log2 Fold Change| >1. Red points indicate metabolites upregulated in the LDL-C target-achieved (L-70, LDL-C < 70 mg/dl) group (n = 118), while blue points represent those downregulated (n = 21). Grey points denote non-significant features.

Candidate metabolites were initially prioritized based on a VIP score >1.4 and confirmed with Level half annotation. To rigorously validate their independent associations with LDL-C target achievement, we applied multivariable linear modeling with MaAsLin2 (24), adjusting for age, sex, CKD status, and ACEI/ARB use. The adjusted model confirmed significant between-group differences for eight key target metabolites. Specifically, the LDL-C target-achieved group had independently lower fecal levels of eight amino acid-related metabolites: glutamine, glutamate, phenylalanine, N-acetyl-L-phenylalanine, L-methionine, N-acetyl-L-methionine, lysine, and N-methyl-D-aspartic acid (Fig. 4 and supplemental Table S1). Furthermore, caffeine and valsartan also retained significant differences in the adjusted model, suggesting potential exposure- or medication-related signatures detectable in stool metabolomics.

**Fig. 4:**
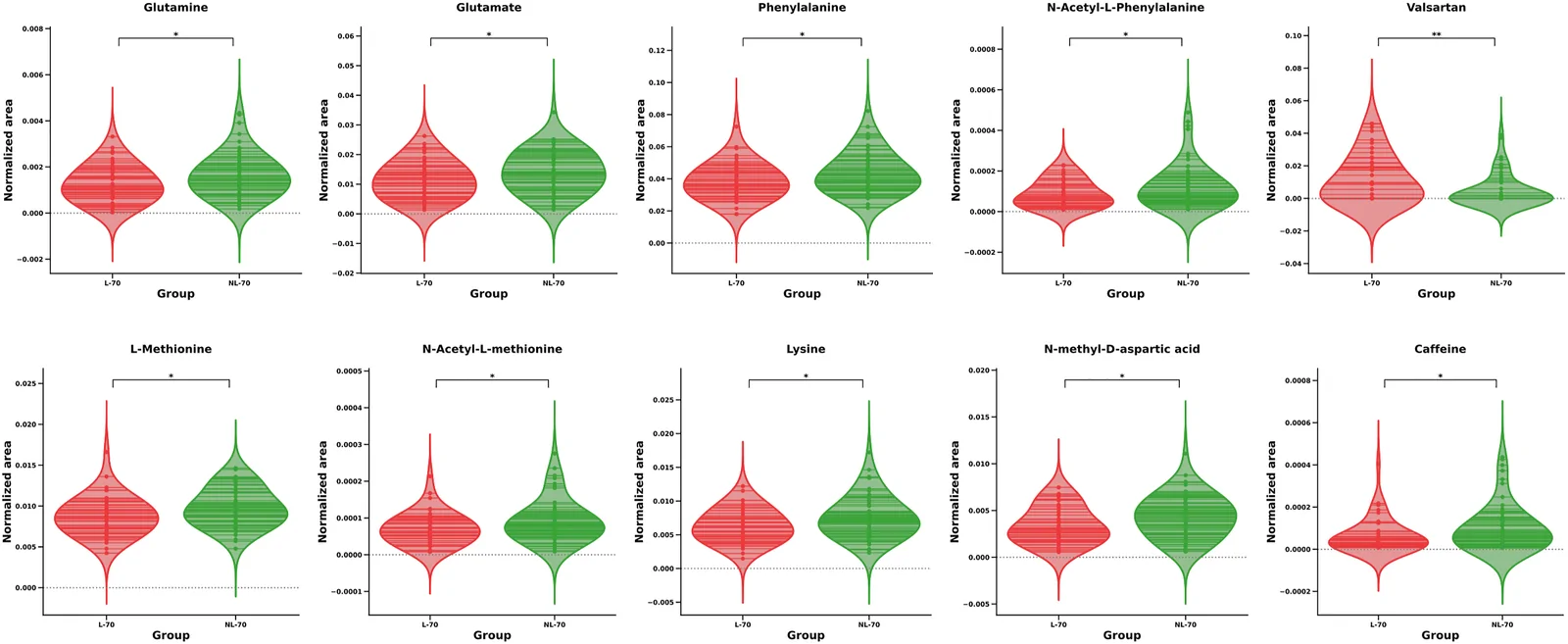
Key fecal metabolites by LDL-C target achievement status. Violin plots showing normalized abundance levels of significant metabolites in the LDL-C target-achieved group (L-70, red, n = 52) versus the target-not-achieved group (NL-70, green, n = 72). Individual samples are overlaid within each violin. Data are presented as distribution density with median markers. Group comparisons were performed using a two-tailed Student’s *t* test; significance is indicated as *P* < 0.05 (∗) and *P* < 0.01 (∗∗). The asterisks denote nominal (unadjusted) *P*-values from univariate two-tailed t-tests and are distinct from the FDR-adjusted MaAsLin2 multivariable analysis used to identify the independently associated metabolites (supplemental Table S1).

### Pathway and metabolite set enrichment analysis

Metabolite set enrichment analysis highlighted pathways predominantly related to amino acid and nitrogen metabolism (supplemental Fig. S1). The top enriched sets included cysteine and methionine metabolism, lysine degradation, arginine biosynthesis, arginine and proline metabolism, phenylalanine metabolism, and alanine/aspartate/glutamate metabolism, as well as redox- and energy-related pathways including glutathione metabolism, and SCFA-related pathways including butanoate metabolism and propanoate metabolism (supplemental Fig. S1). Collectively, these findings suggested that not achieving the LDL-C target was associated with the enrichment of fecal metabolite patterns related to amino acid handling and downstream metabolic network changes.

### Co-occurrence modules of gut microbes and target metabolites

To investigate the potential microbial origins of the altered fecal amino acid profiles, we integrated 16S rRNA microbiome and metabolome datasets using the paired subset (n = 86). Following our prior characterization of distinct gut microbiota profiles associated with LDL-C target achievement (9), we sought to elucidate the functional microbe-metabolite interplay. Hierarchical clustering of the Spearman correlation matrix across all detected taxa revealed a structured regulatory module. Within this network, specific bacterial genera, and most notably *Anaerotruncus*, *UBA1819*, and *Dielma*, emerged as strongly and positively correlated (*P* < 0.05, r >0.5) with key differential metabolites, including lysine, glutamate, and N-methyl-D-aspartic acid (supplemental Fig. S2). Conversely, *Acidaminococcus* and *Psychrobacillus* were inversely correlated with phenylalanine derivatives. Collectively, these multiomics associations suggested a specialized microbial modulation of the fecal amino acid pool linked to LDL-C target achievement.

### Network centrality and the aanaerotruncus–amino acid hub

Topological analysis of the multiomics network (*|r|* >0.35) identified *Anaerotruncus* as a primary hub microbe with high degree centrality (Fig. 5). Convex hull delineation encapsulated the “Core Metabolic Hub,” representing the dense interaction zone between *Anaerotruncus* and its associated amino acid cluster (e.g., glutamine, lysine). This topological arrangement highlighted the biological relevance of the *Anaerotruncus*–amino acid axis in host metabolic status, distinguishing this hub from peripheral nodes such as *Bacteroides* and *Faecalibacterium*.

**Fig. 5:**
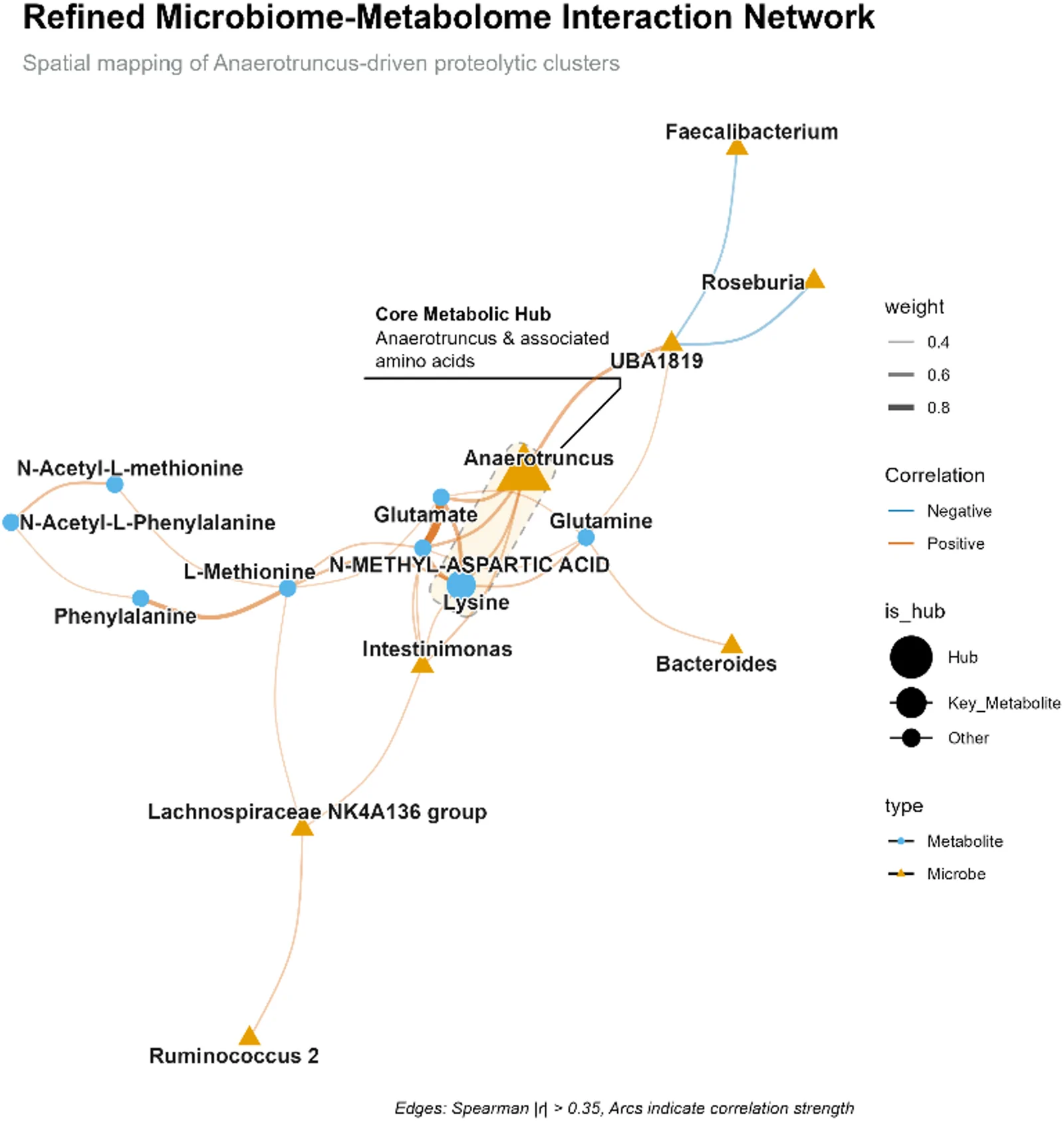
Network visualization of the Anaerotruncus-centered metabolic hub. Interaction network illustrating significant associations between gut microbes (orange triangles) and fecal metabolites (blue circles). Edges represent Spearman correlations (|r| > 0.35), with orange and blue curves indicating positive and negative associations, respectively. The node size is proportional to centrality. The shaded region (convex hull) defines the “Core Metabolic Hub,” emphasizing the dense functional connectivity between *Anaerotruncus* and key amino acid derivatives.

## Discussion

In this cross-sectional study of 124 statin-treated patients with chronic diseases, we identified a distinct fecal metabolomic profile associated with LDL-C target achievement (LDL-C <70 mg/dl). The target-achieved group had consistently lower fecal levels of eight annotated metabolites—glutamine, glutamate, phenylalanine, N-acetyl-L-phenylalanine, L-methionine, N-acetyl-L-methionine, lysine, and N-methyl-D-aspartic acid—which remained significant after multivariable adjustment. Pathway enrichment analysis further supported a predominant amino acid and nitrogenbmetabolism signature. Integrative analysis using paired 16S rRNA data (n = 86) highlighted a structured microbe–metabolite correlation network, with *Anaerotruncus* emerging as a central hub linked to amino-acid-related metabolites. Collectively, these findings suggested that intestinal nutrient handling, and particularly amino-acid availability and related microbial metabolic pathways, were associated with the degree of LDL-C lowering achieved under statin therapy.

### A “low fecal amino acid” signature may reflect a more favorable intestinal metabolic state

An important finding of this study is the concordant reductions in multiple amino acids and related derivatives in stool samples of those who met the LDL-C target. Fecal amino-acid levels represent a composite of dietary protein intake, host digestion and absorption, endogenous protein secretion, and microbial uptake and transformation (25, 26, 27, 28). Lower levels of fecal amino acids may therefore indicate: (i) more efficient host absorption and utilization (26), (ii) greater microbial incorporation into biomass (29, 30), and/or (iii) reduced proteolytic fermentation and amino-acid spillover into the distal colon (31, 32). Microbial amino-acid metabolism is increasingly recognized as a modulator of host metabolic health, and gut bacteria have been shown to alter amino-acid bioavailability and generate downstream metabolites that can influence inflammation, gut barrier function, and systemic cardiometabolic risk (33). In this context, a coordinated decrease in glutamine/glutamate, methionine, lysine, and phenylalanine-related metabolites may represent a gut ecosystem with reduced excess luminal amino-acid substrate and a shift away from a “protein-fermentation dominant” pattern.

### Glutamine/glutamate and intestinal barrier considerations

Glutamine and glutamate are biologically notable because glutamine is a major energy substrate for intestinal epithelial cells, and it has been implicated in the regulation of tight junction integrity (34, 35). A study involving an intestinal epithelial model showed that glutamine deprivation could reduce transepithelial resistance and increase permeability via effects on tight junction proteins (36). While our cross-sectional fecal data cannot establish the mechanism, lower fecal glutamine/glutamate in the LDL-C target achievers could be consistent with enhanced epithelial utilization or altered microbial uptake, potentially aligning with a more intact barrier milieu and less luminal amino-acid accumulation (28, 34, 37).

### Lower fecal phenylalanine/N-acetyl-L-phenylalanine and a potentially “less pro-thrombotic” phenylacetylglutamine axis

The lower fecal phenylalanine level in those who achieved the LDL-C target of <70 mg/dl may reflect reduced colonic aromatic–amino-acid spillover (better host absorption and/or less proteolytic fermentation), which would plausibly limit microbial generation of phenylacetic acid (PAA)—a key upstream precursor feeding host conjugation to PAGln (38). Because PAGln has been mechanistically linked to enhanced platelet activation and thrombosis via adrenergic receptors and is clinically associated with cardiovascular risk, a lower fecal phenylalanine signal is directionally consistent with a gut metabolic milieu that could be less pro-thrombotic—pending confirmation with paired plasma PAGln/PAA measurements (38). The concurrent reduction of N-acetyl-L-phenylalanine may further support altered aromatic–amino-acid nitrogen handling, as acetylated amino-acid derivatives have been discussed in the broader uremic-solute/cardiometabolic risk framework, which is particularly relevant in cohorts with a high prevalence of CKD (39, 40). Notably, stool depletion alone cannot distinguish decreased production from increased microbial assimilation or increased host uptake, and therefore integrating metagenomic pathway quantification (PAA/PAGln biosynthetic capacity) and plasma metabolomics is necessary before inferring downstream cardiovascular benefits (41, 42).

### Microbe–metabolite network: anaerotruncus as a potential hub taxon

Microbe–metabolite network analysis highlighted *Anaerotruncus* as a potential hub taxon that was positively correlated with lysine, glutamate, and N-methyl-D-aspartic acid, whereas several other taxa showed inverse associations with phenylalanine-related metabolites (Fig. 5). *Anaerotruncus* (including *Anaerotruncus colihominis*) was originally described as a human gut obligate anaerobe capable of producing acetate and butyrate, placing it in an SCFA-associated ecological niche that is broadly linked to intestinal barrier and immunometabolic homeostasis (43, 44). Consistent with this functional framing, a prior multiomics study reported that *Anaerotruncus* was associated with variations in host amino-acid–related metabolic modules (including lysine-linked signatures), supporting the interpretation that an *Anaerotruncus*-centered consortium may be associated with luminal nitrogen handling and amino-acid availability rather than implying a single direct biosynthetic role (45). Mechanistically, butyrate formation in the gut can arise not only from carbohydrate fermentation but also from amino-acid–derived routes, including pathways fed by lysine, and these processes often involve community-level cross-feeding rather than one organism acting in isolation (46, 47, 48). Importantly, “hub” behavior in correlation networks is hypothesis-generating and does not establish causality; moreover, genus-level 16S profiles cannot resolve strain-level functions. Nevertheless, the observed alignment of *Anaerotruncus* with amino-acid–related metabolites may be relevant in statin-treated individuals, because accumulating evidence has indicated that the gut microbiome can modulate statin lipid responses (including LDL-C lowering), and statin exposure itself has been associated with shifts in gut microbial and metabolomic profiles in human and experimental studies (49, 50, 51). In contrast, direct functional links between *Anaerotruncus* and N-methyl-D-aspartic acid remain insufficiently characterized; thus, we interpret this association conservatively as reflecting a broader amino-acid–derivative pool that covaries with local fermentation ecology (52).

### Strengths and limitations

This study has several strengths, including a clinically relevant statin-treated cohort, robust untargeted fecal metabolomics with stringent annotation filters for prioritized metabolites, multivariable modeling to address key confounders (including CKD status and ACEI/ARB use), detailed ascertainment of statin exposure and of nine classes of glucose-lowering therapy, and a paired microbiome–metabolome integrative analysis framework. However, limitations should be acknowledged. First, the cross-sectional design precludes causal inference; differences in fecal metabolites may reflect pre-existing gut metabolic states rather than consequences of LDL-C control. Second, although statin agent, dose, dosing frequency and treatment duration and nine classes of glucose-lowering therapy—including metformin and α-glucosidase inhibitors, which are known to modify the gut microbiota and fecal metabolome—were ascertained in detail and were comparable between the groups, dietary intake and objective measures of adherence were not available and could influence fecal amino-acid pools and LDL-C levels. Once-daily dosing was more common among target achievers, so a contribution of dosing pattern cannot be entirely excluded. Third, fecal metabolomics reflects luminal content and may not mirror systemic exposure; paired plasma metabolomics and targeted quantification of key microbial co-metabolites (e.g., PAGln), bile acids, sterols, and SCFAs are needed. Fourth, microbiome profiling was available in only a subset of patients and was based on 16S rRNA sequencing at genus-level resolution; functional inference remains limited without shotgun metagenomics and experimental validation. A further consideration is that the LDL-C threshold of <70 mg/dl used to define target achievement corresponds to the risk category represented by the present cohort rather than to the <55 mg/dl goal recommended for very high-risk patients with established atherosclerotic cardiovascular disease; whether the fecal metabolome differs further below that lower threshold warrants dedicated study. Finally, residual confounding by concomitant medications and comorbidity burden remains possible despite adjustments.

### Conclusion

Among statin-treated adults, LDL-C target achievement was associated with a coherent “low fecal amino acid” metabolomic signature and enrichment of amino-acid and nitrogen-related metabolic pathways. Integrated microbe–metabolite analysis suggested that specific taxa, and particularly *Anaerotruncus*, may participate in ecological modules linked to these metabolite patterns. These findings support the concept that intestinal nutrient metabolism and gut microbial ecology are associated with variability in LDL-C control in individuals receiving statin therapy. Further prospective, mechanistic studies integrating targeted metabolomics, metagenomics, and clinical interventions are warranted to clarify causality and translational utility.

## Data availability

All data supporting the conclusions of this study are presented in the article and in the supplemental data. The untargeted fecal metabolomics data sets (raw files and processed feature tables) and the 16S rRNA gene sequencing data generated in this study are available from the corresponding author (Szu-Chia Chen, scarchenone@yahoo.com.tw) upon reasonable request; access to individual-level participant data is restricted by the data-sharing conditions of the Institutional Review Board of Kaohsiung Medical University Hospital.

## Supplemental data

This article contains supplemental data (supplemental Table S1 and supplemental Figs. S1 and S2).

## Conflict of interest

The authors declare that they have no conflicts of interest with the contents of this article.
