# Supplementary material for: Fecal metabolomic profiles by LDL-C target achievement status in statin-treated patients: a cross-sectional study
Source: J Lipid Res. 2026 Sep 1;67(10):101136. doi: 10.1016/j.jlr.2026.101136 (PMC13635716; doi:10.1016/j.jlr.2026.101136)
Supplement: Supplementary file 1 [file mmc1.docx]

# Supplemental Information

## Figure S1: Overview of the top 25 enriched metabolic pathways stratified by LDL-C target achievement. Dot plot showing significant metabolite sets identified via MSEA. Pathways are ranked by significance (-log10 p-value), with dot size representing the enrichment ratio and color gradients indicating statistical significance (red: more significant, i.e., lower p-value; orange: less significant).

## Figure S2: Correlation matrix of key gut microbes and fecal metabolites. Heatmap displaying Spearman’s rank correlations (r) between selected bacterial genera and metabolites. Color intensity indicates the strength and direction of association (Red: Positive; Green: Negative). Black-bordered cells indicate statistical significance (p < 0.05). Clustering revealed specific microbial modules associated with distinct metabolic profiles.

**Table S1. Adjusted multivariable associations of fecal metabolites with LDL-C target achievement**. MaAsLin2 multivariable modeling of fecal metabolites associated with LDL-C target achievement, adjusted for age, sex, CKD, and ACEI/ARB use.

| **Taxa** | **coef^[1]^** | **stderr^[2]^** | **N^[3]^** | **N**  **not 0^[4]^** | **p-value** | **q-value** |
| --- | --- | --- | --- | --- | --- | --- |
| Glutamine | 0.548 | 0.208 | 124 | 124 | 0.010 | 0.060 |
| L.Methionine | 0.149 | 0.064 | 124 | 124 | 0.022 | 0.060 |
| Lysine | 0.233 | 0.108 | 124 | 124 | 0.033 | 0.060 |
| Phenylalanine | 0.156 | 0.074 | 124 | 124 | 0.036 | 0.060 |
| N.Acetyl.L.Phenylalanine | 0.472 | 0.217 | 124 | 124 | 0.032 | 0.060 |
| N.Acetyl.L.methionine | 0.414 | 0.186 | 124 | 124 | 0.028 | 0.060 |
| Glutamate | 0.330 | 0.173 | 124 | 124 | 0.059 | 0.066 |
| N-methyl-D-aspartic acid | 0.333 | 0.166 | 124 | 124 | 0.048 | 0.066 |
| Caffeine | 0.462 | 0.269 | 124 | 124 | 0.089 | 0.089 |
| Valsartan | -1.454 | 0.762 | 124 | 124 | 0.059 | 0.066 |

1. The model coefficient value (effect size)
2. The standard error from the model
3. The total number of samples used in the model
4. The total of number of these samples in which the feature is non-zero We could do a check of the email.
